# Autonomy-hindering scope for physiotherapy practice in African countries: Results of creatures and antinomies of regulatory laws

**DOI:** 10.4102/sajp.v77i1.1518

**Published:** 2021-02-25

**Authors:** Ushotanefe Useh

**Affiliations:** 1 Lifestyle Diseases Research Entity, Faculty of Health Sciences, North-West University, Mmabatho, South Africa

**Keywords:** physiotherapy, autonomy-hindering laws, Africa, jurisdictional scope, regulatory, legal, statuses

## Abstract

**Background:**

Healthcare professionals in different countries are governed by laws and statutes for their scopes of practice to ensure that services are rendered by suitably licenced and qualified professionals in order to protect the public. A few of these laws are found to paradoxically hinder the autonomy of physiotherapy.

**Objective:**

My article documents the autonomy-hindering scope for physiotherapy practice in selected African countries.

**Method:**

The methodologies used in my article were both a review and comparative approach for the interpretation of statutes.

**Results:**

Three African countries presented a clear legal definition of physiotherapy in their regulatory frameworks and regulated other rehabilitation professions as well. In my article, these regulations are referred to as ‘combo regulations’. The rationale for ‘combo regulations’ is not clear and found to hinder professional autonomy. Only one statute from Rwanda provided a scope for physiotherapy that was not autonomy-hindering.

**Conclusion:**

There is, therefore, a need for urgent review of most laws regulating physiotherapy in the selected African countries to assist with the duty of protecting the public. All autonomy-hindering scopes for physiotherapy practice in African countries should be repealed and amended accordingly.

**Clinical implications:**

A clear scope shall assist with protecting the public and clinical practice and clearly states ‘what physiotherapy is and what it is not’.

## Introduction

The purpose of *Healthcare Practice Acts* includes, amongst others, the enacting of regulations and laws that enable various health professions to define their areas of expertise, as delineated in their scope of practices and statutes. The scope of practice is a way of describing what you are trained and competent to do. It describes the areas in which you have the knowledge, skills and experience to practice safely and effectively in the best interests of patients (General Dental Council 2009). This assertion is also supported by Kersten et al. (2007) and the General Dental Council (2009) who stipulate that the scope of practice is likely to change throughout our careers and that this may expand to developing new skills or may narrow and deepen the knowledge of a particular area. The dynamism in ‘Extended Scope Physiotherapists’ as clinical specialists to develop and demonstrate expertise beyond the currently recognised scope of practice, including some aspects of job enhancement or expansion, involving the areas of extended therapeutics, also follows the same argument (Stanhope et al. 2012). Legislation that recognises physiotherapy as an autonomous profession, able to accept patients via direct access and self-referral, is perceived as a significant facilitator and as a barrier when it is absent (Bury & Stokes 2013b). Certainly, if legislation is introduced in those countries currently without it, it will be important to retain these professional autonomy roles (Bury & Stokes 2013a). Different regulatory authorities assist with the enforcement of the individual profession’s scope of practice to ensure that healthcare services are provided by suitably licenced and/or certified and qualified professionals. The practice of physiotherapy in many countries is protected by laws or statutes in accordance with the policy of the World Confederation for Physical Therapy (WCPT). However, the enactment of laws governing the practice is left to different countries (WCPT 2017), according to the policy statement of the WCPT (2017).

WCPT (2017) indicates that:

[*T*]he profession of physical therapy is responsible for the articulation of the profession’s scope of practice and defining the roles of physical therapists. (pp. 1–6)

This scope of practice is well defined in the United States, Canada, New Zealand and Australia and governs the practice of physiotherapy as a separate profession in the healthcare industry ([APTA] American Physical Therapy Association 2020). Meanwhile, many countries on the African (except South Africa and Rwanda) and Asian continents do not have clear and well-defined jurisdictional scopes for the practice of physiotherapy. In some instances, like the one in South Africa, it is also autonomy-hindering as the laws governing the scope of physiotherapy practice are supplementary to medicine (Regulations Defining the Scope of the Profession of Physiotherapy 1976).

The WCPT (2017) policy clearly defines the scope of practice for physical therapy or physiotherapy which amongst others, states that physical therapy is a dynamic profession with an established theoretical and scientific base and widespread clinical applications in the restoration, maintenance, and promotion of optimal physical function. WCPT (2017) further asserts that physical therapists or physiotherapists are health care professionals who help individuals maintain, restore, and improve movement, activity, and functioning, thereby enabling optimal performance and enhancing health, well-being, and quality of life. Their services prevent, minimize, or eliminate impairments of body functions and structures, activity limitations, and participation restrictions. According to WCPT (2017), national physical therapy/physiotherapy associations are responsible for defining physiotherapy and its role which should be both relevant to their nation’s health delivery needs and be consistent with acceptable international guidelines. (pp. 1–6)

However, it is worth noting that the WCPT documents are not juristic documents but guidelines and therefore not legally binding and cannot be used as a source of law in court (WCPT 2017). Therefore, it is expected that different countries should enact laws at a national level to delineate and protect the practice of physiotherapy.

In South Africa, and as in many countries, the recognised sources of laws are the constitution, which is prescribed by Section 2 of the Constitution of the Republic of South Africa (Constitution of the Republic of South Africa 1996) as the supreme law of the land. Other sources of laws are statutes or legislation and decided cases. A statement of the law itself from a governmental entity, such as a court, legislature, the executive arm of government, president or governor or premier (as in South Africa) are all considered primary sources of law. These primary sources of law have binding effects, whilst secondary sources of laws such as textbooks, legal journal articles and writings have a persuasive effect, that is, they assist in discussing, explaining, interpreting and analysing what the law is, or what it should be (Botha 2012). Therefore, the WCPT guideline that describes the scope of physical therapy or physiotherapy has no binding effect because it is not a primary source of law but a guiding document.

In many African countries, there is an autonomy-hindering scope of physiotherapy practice. According to Sandstrom (2007), professional autonomy is a ‘social contract based on public trust in an occupation to meet a significant social need and to preserve individual autonomy’. Professional autonomy includes control over the decisions and procedures related to one’s work (technical autonomy) and control over the economic resources necessary to complete one’s work (socio-economic autonomy).

This article sought to document the current jurisdictional description or legal definition of physiotherapy as a profession not hindered by its laws in selected African countries. There is an urgent need to clearly document the jurisdictional scope for physiotherapy in these countries and to amend these laws as they hinder the autonomy of physiotherapy (Regulations Defining the Scope of the Profession of Physiotherapy 1976) and to avoid possible litigation on the definition of physiotherapy as reported in the case of the *South African Society of Physiotherapy v Equine Librium College* and *South African Society of Physiotherapy v Equine Librium College and Others 2017.* In these cases, physiotherapy was referred to as a definitive word and treated as a ‘passing-off’ case. According to Klopper et al. (2011), ‘passing off’ occurs when a trader or entity uses the distinctive marks (trademark or trade names) of a competitor to create the impression that his or her performance is similar to the competitor’s well-known performance, thereby deceiving consumers into accepting his or her performance. This is used by competitors to coax consumers unfairly and unlawfully.

There is a need to legally demonstrate that physiotherapy has acquired a distinctive character or a secondary meaning such that the descriptive name can be protected.

## The operational definition of terms

### Jurisdiction

This is generally defined as the right, power or authority to administer justice by hearing and determining controversies. However, in my article, the jurisdictional (i.e. legal) scope of practice is established by a state’s practice act governing the specific physical therapist’s (physiotherapist’s) licence, and the rules adopted in accordance with that act (APTA 2020).

### Regulation

Rule made and maintained by a designated authority.

### Scope of practice

The professional scope of practice of physical therapy (physiotherapy) is defined as a practice that is grounded in the profession’s unique body of knowledge, supported by educational preparation, based on a body of evidence, and linked to existing or emerging practice frameworks (APTA 2020).

### Personal scope of physical therapist practice

Personal scope of practice consists of activities for which an individual physical therapist (physiotherapist) is educated and trained and that he or she is competent to perform (APTA 2020).

### Passing off

Passing-off occurs when a trader or entity uses the distinctive marks (trademark or trade names) of a competitor to create the impression that his or her performance is similar to that of the competitor’s well-known performance, thereby deceiving consumers into accepting his or her performance.

### Antinomies

Paradox.

## Methodology

The methodologies used in my article were both a review and a comparative approach of interpretation of laws or statutes according to Botha (2012). Botha refers to five interrelated dimensions of interpretation of statutes.

Amongst these are language, systematic, holistic (contextual and structural), teleological or a value-laden dimension, historical and comparative dimensions. The primary rule of interpretation is the application of the plain or literal meaning of the statute; but if the ‘plain meaning’ of the words is ambiguous, vague or misleading, the courts consider the wider context of surrounding circumstances, giving rise to the golden rule or recourse to the mischief that the statute was to curb, or other rules of interpretation. The interpretation of statutes is supported by section 39 (1) of the Constitution of South Africa (Constitution of the Republic of South Africa 1996). It states that when interpreting the Bill of Rights, a court, a tribunal or a forum: (1) must promote the values which underlie an open and democratic society based on human dignity, equality and freedom; (2) must consider international law; and (3) may consider foreign law.

The three components of the scope of practice for physical therapists (physiotherapists) as described by APTA served as a guide for analysing the scope of practice in my article (APTA 2020). This guide was chosen because it was the most comprehensive legal approach found in the literature.

These three components are (1) the professional scope of physical therapist (physiotherapist) practice, (2) jurisdictional (legal) scope of physical therapist (physiotherapist) practice and (3) personal scope of physical therapist (physiotherapist) practice. The professional scope of practice of physical therapy (physiotherapy) is defined as a practice that is grounded in the profession’s unique body of knowledge, supported by educational preparation, based on a body of evidence, and linked to existing or emerging practice frameworks.

Jurisdictional scope is generally defined as the right, power or authority to administer justice by hearing and determining controversies. However, in my article, the jurisdictional (i.e. legal) scope of practice is established by a state’s practice act governing the specific physical therapist’s (physiotherapist’s) licence and the rules adopted in according to that act (APTA 2020). Whilst the personal scope of practice consists of activities undertaken by an individual physical therapist (physiotherapist) that are situated within a physical therapist’s (physiotherapist’s) unique body of knowledge where the individual is educated and trained and becomes competent to perform that activity.

### Regulatory statutes selected

Statutes regulating physiotherapy from eight African countries were purposively selected for review of professional, personal, and jurisdictional scope of physiotherapy practice. The selected countries of Ghana, Nigeria, Namibia, Kenya, Rwanda, South Africa, Zambia and Zimbabwe are also active members of WCPT. Their regional distributions are sub-Saharan, West, Southern and East Africa.

### Ethical consideration

My article followed all ethical standards for research without direct contact with human or animal participants.

## Results and discussion

### The legal definition of physiotherapy in the selected African countries

I found no legal definitions of physiotherapy in the statutes regulating the practice of physiotherapy in many of the African countries studied. Also, there is no mention of a clear scope of professional practice in the statutes that govern physiotherapy in many African countries. The lack of a clearly defined professional scope of practice in some of these African countries might be because of the fact that most laws governing the practice of physiotherapy in Africa (except South Africa, Rwanda and Kenya) are also enacted for a group of other professions (referred to as combo-legislation) with the erroneous assumption that all these professions, with a very different professional and theoretical body of knowledge base, are the same. Therefore, developing a professional scope for these would be too comprehensive and almost impossible.

### Autonomy-hindering scope

All but one was found to be an autonomy-hindering scope. The reasons for this might be historical and probably linked to how these professions evolved. This agrees with the disclosures of Loh et al. (2017), who found a common feature of less developed countries codifying healthcare within an entrenched autocratic medical model, compared to a more autonomous model of health services in more developed countries. Under such medical governance, occupational therapists often experience a lack of empowerment to implement occupational therapy interventions independently, develop new evidence-based programmes or make decisions in collaboration with their patients – an experience that meets the definition of occupational injustice and denial of rights to engage in occupations that meet the individual needs of occupational therapists and their capacity to develop their potential. This results in a constrained autonomy.

We are free in all spheres of our lives, including professions, when we are free to make decisions and act upon them (Lau & Wenzel 2015). The profession of Speech and Language Pathologists (SLP) stipulates in its scope the need for professional autonomy. According to the American Speech-Language-Hearing Association (2016), SLPs are autonomous professionals who are the primary care providers of speech-language pathology services. Speech and language pathology services are not prescribed or supervised by other professionals. This is in variance to the 1976 scope of physiotherapy in South Africa which makes it a supplementary service to medicine, therefore legally losing its autonomy. The South African scope further reiterates that:

The following acts are hereby specified as acts which shall for the Act be deemed to act about the profession of physiotherapy. These acts shall be performed in the following fields covered by physiotherapy as a supplementary service to medicine. (Regulations Defining the Scope of the Profession of Physiotherapy 1976:1–8)

This Act denies physiotherapy any form of professional autonomy as it further includes the following:

‘[*P*]hysiotherapeutic examination of patients according to the condition diagnosed by the medical practitioner or dentist, including continuous assessment of the patient’s response to physiotherapy treatment and progress made. Such examination includes the assessment of joint range; muscle power, strength, tone, endurance, and coordination, righting, balance and equilibrium reactions; postural abnormalities, functional ability, the need for rehabilitation and degree of independence attained …’. (Regulations Defining the Scope of the Profession of Physiotherapy 1976:1–8)

**FIGURE 1:**
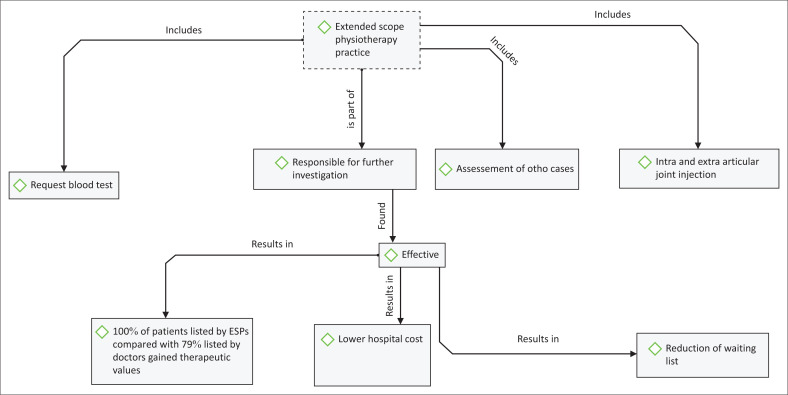
Impact of extended scope of physiotherapy in patients’ care.

The assertion above will in no small measure hinder professional growth. Dawson and Ghazi (2004) reported that in 1999, a randomised controlled trial comparing extended scope practitioners (ESPs) to post-fellowship junior staff using similar sized samples showed that the former were as effective as medical doctors in the initial assessment and management of new referrals to the orthopaedic clinic, generating fewer hospital costs with statistically higher patient satisfaction rates (Figure 1). Further evidence revealed an accurate assessment by physiotherapists, comparing the accuracy of clinical diagnosis with arthroscopy findings between an ESP, consultant and sub-consultant doctors through a 5-month retrospective audit found that clinical diagnosis matched surgical findings in 52% of patients referred by ESP compared to 37% of their medical counterparts (Gardiner & Turner 2002). Also, a randomised controlled trial, comparing ESPs with sub-consultant surgeons in the initial assessment and management of General Practitioners referrals to outpatient orthopaedic departments, found that ESPs incurred lower hospital costs (Gardiner & Turner 2002). Arthroscopies were deemed of therapeutic value in 100% of ESPs referrals compared to 79% listed by doctors (Dawson & Ghazi 2004). The reason for this is not clear.

According to Ojha, Snyder and Davenport (2014), the skill levels and the training of physiotherapists and their competency are sufficient for them to function in a direct access capacity.

The introduction of extended scope elsewhere allows for continuous professional development, hence the improvement of skill (Morris et al. 2014). Other countries are attempting to blur the professional divide in the interest of our patients and healthcare. Hattam (2004) reported that the National Health Service (UK) employers will be required to empower appropriately trained nurses, midwives and therapists to undertake a wider range of clinical tasks, including the right to make and receive referrals, admit and discharge patients, order investigations and diagnostic tests, run clinics and prescribe drugs. This might have an impact on professional autonomy or access to treatment. Factors that might influence professional autonomy and access to physiotherapy in Africa are recommended for further investigation. The power of the medical professionals and the power of politicians were also documented as possible facilitators and barriers to direct access (Bury & Stokes 2013a).

Professional autonomy was embedded in the Rwandan regulations of referral with the option of referral from medical officers. This was the only statute that was found not to be autonomy-hindering for physiotherapy practice in Africa. Also, it provided a jurisdictional definition for physiotherapy within its statute, as in Article 40 of the Ministerial Order No 20/24 of 2011 (Rwanda Physiotherapist Article 40 2011). This regulatory statute also clearly stipulates a hierarchical model for others in the rehabilitation profession.

There was neither a clear definition of the profession of physiotherapy, nor the scope of practice of physiotherapy in the statutes that were enacted to regulate physiotherapy in Nigeria (Medical Rehabilitation Therapists Act 1988) and Kenya (Kenya Physiotherapy Act 2014).

One may argue that a clearly defined scope might eliminate ambiguity when professional conflict arises. In South Africa, the regulations defining the scope of the profession of Biokinetics ‘as published under government notice R1746 in the Government Gazetted 1602 of the Health Professions Council of South Africa (1974)’ defines biokinetics as the profession concerned with:

[*P*]reventive health care, the maintenance of physical abilities, and final phase rehabilitation, using a scientifically-based physical programme. (Regulation Defining The Scope of the Profession of Biokinetics 1994:1–3)

The above is similar to and within the scope described by the different physiotherapy regulatory bodies including the one followed by the South Africa Society of Physiotherapy (SASP) which is:

[*A*]ssessing, treating and preventing human and animal movement disorders, restoring normal function or minimising dysfunction and pain in adults and children with physical impairment, to enable them to achieve the highest possible level of independence in their lives; preventing recurring injuries and disability in the workplace, at home, or during recreational activities and promoting community health for all age groups. (South African Society of Physiotherapy 2019)

This lack of clarity creates possible conflicts between professions and confusion amongst members of the public, including medical doctors and other members of the health professions on the different roles and scope of practice by physiotherapists and biokineticists. A clear demarcation of scope between these two disciplines will better serve the interest of the public and patients. In *Kleuver v de Goede* (Kluever v de Goede 2015), Dr. Kluever in para 36 had indicated that the failure of the patella to heal was because of strenuous exercises by the athlete on the advice of the physiotherapist and biokineticist (which they both denied). If there is a scope for clarity between these professions, the medical doctors would be able to refer patients appropriately. A similar conflict was also reported in *Hall v Thomas*, (Hall v Thomas and others 2014) between the coach and fitness trainer in the final phase of rehabilitation whilst the contributory role of the physiotherapist in sports rehabilitation was established by the court.

The courts or alternate dispute resolution will always be approached to resolve conflicts arising from ambiguities in the scope of different professions. One such case is the case of *SASP* v Equine Librium (South African Society of Physiotherapy v Equine Librium College and Others 2017) where physiotherapy was used by Equine Librium College. This ruling exposes the South African physiotherapist to the risk of intrusion or encroachment by other professions and persons. The court in the above case, whilst referring to *Burnkloof Caterers Pty v Horseshoe Caterers (Pty) LTD* (1976), indicated that:

A trader who uses a descriptive word in designating his business must ordinarily submit to the risk of some confusion arising among the public if another trader uses the same word concerning his business. (p. 403)

This ruling is enough justification for the SASP to make necessary submission to the Health Professions Council of South Africa (HPCSA) for an amendment to the scope of practice of physiotherapy in South Africa. In paragraph 27 of the same judgement, Judge Binns-Wards also stated that:

If the effect of the promulgation under the Veterinary and Para-Veterinary Professions Act of a para-veterinary profession to be called ‘veterinary physiotherapy’ would prejudice the professional status or reputation of the profession regulated under the Health Professions Act, as the plaintiff alleges, that is a matter to be resolved in the first instance between the respective members of the Cabinet responsible for the administration of those Acts, and the engagement of the courts in such matters is something that the Constitution (s 41) and the Intergovernmental Relations Framework Act 13 of 2005 provide should be a last resort. (South African Society of Physiotherapy v Equine Librium College and Others 2017: Para 27)

The South African Society of Physiotherapy’s claim for the protection of ‘physiotherapy’ is not only within the jurisdiction of the court but also clearly embedded in sections 40 b and c of the *Health Professions Act* No. 56 of 1974 as amended (Health Professions Council of South Africa 1974). This is clearly dealt with through the provision of a statute which defines, regulates and delineates the jurisdictional scope of physiotherapy but does not include animal rehabilitation, as was done by APTA (2020), but not The Chartered Society of Physiotherapy (CSP) (2016) because there is no regulation of physiotherapy practice that involves the treatment of animals in the United Kingdom.

In 2008, there was a need to amend the vague scope of practice of the *Health Practitioners Competence Assurance Act* 2003 by the Physiotherapist Board of New Zealand. This was done through a notice of amendment to the scope of practice and related qualifications prescribed by the Physiotherapy Board.

## Conclusion

Different countries have amended the statutes regulating the practice of physiotherapy driven by needs, growth and development of the profession, and other factors. The time is upon us for the representatives of different physiotherapy bodies to approach lawmakers under the ruling of Judge Binns-Wards (as in the case of *SASP v Equine Librium*) and amend the physiotherapy regulations. Borrowing a leaf from other countries, a holistic approach (including *Ubuntu*) could be considered in defining the scope of practice in South Africa, like it was used in the United States stating clearly ‘what physiotherapy is and what it is not’.
